# Harmonized prevalence estimates of dementia in Europe vary strongly with childhood education

**DOI:** 10.1038/s41598-025-97691-z

**Published:** 2025-04-23

**Authors:** Axel Börsch-Supan, Salima Douhou, Marcela C. Otero, Beatrice Baaba Tawiah

**Affiliations:** 1 Max Planck Institute for Social Law and Social Policy, Munich, Germany; 2 Munich Research Institute for the Economics of Aging and SHARE Analyses, Leopoldstrasse 139, 80804 Munich, Germany; 3 National Bureau of Economic Research, Cambridge, MA USA

**Keywords:** Dementia, Cognition, Prevalence, Education, Europe, Cross-national comparisons, Neurological disorders, Cognitive ageing

## Abstract

Up-to-date, strictly cross-nationally comparable and nationally representative data on cognitive health are essential for our understanding of the dementia-related challenges in healthcare, to detect shortcomings in healthcare systems and to design effective prevention strategies. Such data have been missing in Europe. We use the most recent 2022 wave of the strictly harmonized Survey of Health, Ageing and Retirement in Europe (SHARE, 47,773 individuals age 65 and older) to obtain prevalence estimates of mild cognitive impairment and dementia for 27 European countries and Israel in 2022. The novelty of the paper is to validate these estimates using the Harmonized Cognitive Assessment Protocol (HCAP) as a validation tool. These new data exhibit much higher prevalence rates of dementia in the Mediterranean and Southeastern European countries and a much larger variation of cognitive impairment across Europe and Israel than previously known. Dementia prevalence ranges from 4.5% in Switzerland to 22.7% in Spain, MCI prevalence from 17.2% in Sweden to 31.1% in Portugal. Most of this variation can be explained by differences in education when respondents were young. Prevalence rates vary plausibly with other risk factors such as age and comorbidities associated with dementia.

**Supplementary Information:**

The online version contains supplementary material available at 10.1038/s41598-025-97691-z.

## Introduction

The human burden of Alzheimer’s disease (AD) and AD-related dementias (ADRD) is large. According to the most recent Global Burden of Disease (GBD) 2021 report^1^, AD/ADRD is the 8th leading cause of death, and the world-wide economic costs of dementia is estimated to exceed US$1.3 trillion for 55 million people with dementia, corresponding to almost US$24,000 per person with dementia^2^. However, AD/ADRD does not strike countries equally. Both prevalence and costs vary greatly across countries. Understanding these international variations helps to better design national healthcare systems and to reduce the global burden of dementia.

Unfortunately, multi-country prevalence studies of dementia are rare, even for Europe. In addition to GBD, large research consortia have produced prevalence studies of dementia (EURODEM^3,4^, European Collaboration on Dementia (EuroCoDe^5^), Prince et al.^6^, Alzheimer Europe^7^, and the Organisation for Economic Co-operation and Development (OECD^8,9^) which mainly rely on systematic reviews of epidemiological or clinical studies, where inclusion and exclusion criteria are applied to select eligible studies and standardized criteria are used to diagnosis of dementia. Despite strict selection of studies, the studies used are not harmonized in applied methodology, selected population, criteria for diagnosis, age groups covered, study year and other issues that threaten the international comparability of the presented prevalence rates. As population studies have been scarce or missing completely in certain regions (Central and Eastern Europe in particular), it has been difficult to reliably estimate prevalence rates for these countries and missing data had to be imputed from neighboring countries^10,11^. These methodological issues threaten the international comparability of the presented prevalence rates, are an obstacle to obtaining unbiased population estimates and may distort cross-national associations with risk factors for dementia, such as age, sex and education, due to artefacts generated by the lack of comparability across the involved countries.

Significant progress has been made by the Survey of Health, Ageing and Retirement in Europe (SHARE). SHARE is a longitudinal population aging study that started in 2004 and is representative of the 50 + population in 27 European countries and Israel^12^. A key feature of SHARE is the strict ex-ante harmonization of instruments and protocols, including identical cognition measures, which makes it a unique resource for cross-national comparisons of health and socioeconomic status. Based on an earlier wave of these data, a study published in this Journal^13^ has provided dementia prevalence estimates for 2017 using a variant of the Langa-Weir scale of cognitive performance^14^ and compared them to a set of machine learning (ML) based algorithmic approaches. The implementation of ML-based algorithms on SHARE data to assess probable dementia is not new^15–17^. While these SHARE-based studies use strictly comparable indicators across all included countries, they lack a validated approach to identify individuals as MCI or demented. Instead, in one of their approaches, Klee et al. scale their prevalence estimates to national estimates from the OECD^8^ which suffer from the above-mentioned shortcomings.

This paper makes a significant step forward by providing a validation base for the cognition data in the most recent 2022 SHARE wave. We have collected validation data using the international Harmonized Cognitive Assessment Protocol (HCAP). HCAP has been developed by the US Health and Retirement Study (HRS) as part of an international collaboration funded by the National Institute on Aging (NIA) to harmonize the measurement of cognition in a global network of aging studies^18^. SHARE-HCAP is the European arm of the HCAP network of aging studies and includes an in-depth battery of cognitive tests and an informant report on cognitive functioning. We use standard diagnostic criteria to classify respondents into normal, mild cognitive impairment (MCI) and severe cognitive impairment indicating dementia. This classification provides the validation base for our prevalence estimates. Using the classification as a validation base for a larger sample is a significant step forward for the field of dementia epidemiology.Moreover, since standard diagnostic criteria makes our prevalence estimates comparable to those of epidemiological and clinical studies which have been the source for earlier international studies on the epidemiology of dementia, such as^5–9^. A major achievement of the validation is to optimally use all cognition information collected in the full SHARE sample which gives confidence to the prevalence estimates in all 28 SHARE countries. Our validation base and method can be applied to other large scale international studies.

The large neuropsychological battery of HCAP also provides more accurate estimates of mild cognitive impairment, sometimes referred to as CIND (cognitively impaired not demented). Individuals with MCI are at an increased risk of developing dementia as age progresses^19^. Measuring MCI sets our study apart from other recent European-wide studies that focus on dementia diagnosis or are limited to selected countries in Europe. By addressing MCI, we contribute to a comprehensive understanding of preclinical stages of dementia.

Finally, the richness of the combined data allows us to perform face validity assessments by exploring the associations of cognitive performance with the health and socioeconomic characteristics of the individuals in the 28 SHARE countries. SHARE provides information on the most common comorbidities of dementia such as diabetes and cardio-vascular diseases. Moreover, the SHARE data contains an internationally harmonized assessment of the respondents’ educational achievements when they were young (International Standard Classification of Education, ISCED)^20^. Since education early in life exhibits a large variation across Europe, this provides a valuable opportunity to better understand the international variation in cognitive performance and the risk factors for cognitive decline. Our findings suggest that education early in life is a major driver of the international variation in MCI and dementia prevalence. Since education puts individuals on different occupational, economic and lifestyle paths during mid and later life, each with their own effects on cognition, this finding is a potential anchor for preventative measures^21^.

## Methods

Our main data is Wave 9 of the SHARE parent study, which is the most recently available wave of SHARE that took place between October 2021 and September 2022. It includes 47,733 individuals of age 65 and older. SHARE is a nationally representative longitudinal study tracking over time individuals 50 years and older, who have their regular residence in the respective SHARE country and are not incarcerated, hospitalized or out of the country during the survey period and able to speak the country’s language(s). Current partners living in the same household are interviewed as well, regardless of their age. SHARE follows individuals when they move into nursing homes and similar institutions. Probability samples were drawn from population registers in all countries where these were available. SHARE performs proxy interviews for individuals who cannot answer themselves (3.1% in Wave 9, Supplementary Table S2 online). Mortality is ascertained by register checks and followed up by interviews with next of kin to document the final year of life. The SHARE parent study includes indicators of four domains of cognition (memory, executive functioning, language and fluency, and orientation to time and place) that are identical across the 28 countries.

Written informed consent was obtained from all individuals and the SHARE and SHARE-HCAP protocols were approved by the Ethics Committee of the Max Planck Institute in Germany. These studies have been performed in accordance with the Declaration of Helsinki.

Table 1 reports the main sample characteristics of Wave 9. Sample characteristics by country are relegated to the Supplementary Table S1, showing the large differences across countries in terms of age, gender, education, health, and income.

**Table 1:** Sample characteristics of SHARE wave 9 and SHARE-HCAP subsample, weighted^a^.

	SHARE-HCAP subsample (*N* = 2687)	SHARE parent Wave 9 (*N* = 47,733)
Response rate, %	75.8	68.4
Age		
Mean (SD), y	75.5 (7.5)	75.6 (7.7)
Sex		
Female,%	56.2	56.6
Male, %	43.8	43.4
Education (ISCED 1997)		
primary school or less, %	20.0	23.2
Some high school, %	14.9	17.7
High school or some college, %	39.8	37.7
college degree or more, %	25.3	21.4
Health		
ADL + IADL, mean (SD)	0.9 (2.1)	1.2 (2.9)
Household income		
Median in Euro (IQR)	2000 (1700)	1600 (1800)

Total household income per month (average)

ADL, Activities of Daily Living; IADL, Instrumental Activities of Daily Living

We proceeded in four steps detailed below. (a) We drew a validation subsample (*N* = 2,678) of the most recent SHARE wave and administered the neuropsychological battery of SHARE-HCAP in this validation sample. (b) Based on these results, we classified individuals as normal, MCI or demented. (c) We related this classification to those cognition measures that are available in both the SHARE-parent sample and the SHARE-HCAP validation subsample. (d) Using this relation, we predicted for each individual in the analytical SHARE parent sample (*N* = 47,193) the probabilities of cognitive status normal, MCI and demented.

### SHARE-HCAP data collection

SHARE-HCAP collected data on 27 cognitive indicators associated with standard diagnostic criteria (Supplementary Table S3 online) that represent five broad domains of cognition: memory, executive functioning, visuospatial skills, language and fluency, and orientation. These domains were selected based on prior theoretical and empirical work^22^. In addition, a member of the family or a friend was asked to provide an informant’s report.

We selected five countries to represent the East (Czech Republic), West (France and Germany), North (Denmark), and South (Italy) of Europe and drew a weighted subsample of individuals aged 65 years and older from these countries based on the performance in a word recall test in the SHARE parent study, heavily oversampling those with low test scores to ensure an adequate number of individuals who are at a high risk of MCI or dementia.

Data was collected between May and November 2022, on average about five months after Wave 9 of the SHARE parent data collection. Of the 3,546 eligible individuals, 2,687 participated in the SHARE-HCAP study, resulting in an overall response rate of 75.8% (Table 1). They were on average 75.5 (SD = 7.5) years old and primarily female (56.2%). 65.1% completed secondary education as assessed by ISCED. Item nonresponse was low (< 2.3%) except one of the story recall (recognition) (21.9%), the HRS Number Series (15.4%) and TMT part B (12.6%), all three concentrated in Italy. To address this item nonresponse on cognitive measures, we employed Full Information Maximum Likelihood (FIML) estimation in the factor analysis, ensuring that incomplete cases contribute to the estimation process proportionally to their available information. This approach has been shown to produce unbiased parameter estimates and standard errors^23^. Table 1 reports the main sample characteristics of SHARE-HCAP.

### Classification in the SHARE-HCAP sample

For the classification into normal, MCI or demented we followed the approach that has been described in Manly et al.^24^ who relied on diagnostic criteria from the National Institute on Aging and Alzheimer’s Association^25,26^. We choose this approach to allow cross-HCAP study comparisons^27,28^. Details are described in Supplementary Section S1. We first derived factor score estimates of the five domains of cognition for everyone and used a normative sample to set a benchmark for classification. We then classified individuals as demented when the factor scores of at least two cognitive domains were 1.5 SDs below the mean of the normative sample and functional impairment was reported by an informant. Individuals with only one cognitive domain below this threshold and an informant report of functional impairment were classified as MCI, as well as individuals with at least one cognitive domain below the threshold but without an informant report of functional impairment. Moreover, individuals with one cognitive domain below the threshold, no informant report of functional impairment but a self-report of memory concerns were also classified as MCI. Finally, individuals who did not meet the criteria for cognitive impairment in any domain were classified as normal, as well as individuals with one cognitive domain below the threshold but neither a self-report nor an informant-report of cognitive concerns.

### Relation between cognitive status in the SHARE-HCAP sample and cognition measures in the SHARE parent sample

We then related the outcome of the classification described in Step (b) to a selection of demographic variables and cognitive and health measures that are available both in the SHARE-HCAP validation sample and the SHARE parent study using the regression-based approach developed by Hurd et al.^29^ to our multi-country setting. Details are provided in Supplementary Section S2 and Supplementary Table S5. Cognitive items included orientation in time, immediate and delayed word recall, serial 7s, and animal naming (Supplementary Table S4). Health was measured by the sum of activities of daily living (ADL) and the sum of instrumental activities of daily living (IADL). This approach effectively weighs the cognition items of the SHARE parent study by their weights in the SHARE-HCAP sample. Table 2 shows that the prediction by our regression model replicates the classification results from Step (b) very well.

**Table 2:** Estimated prevalence of normal, MCI and dementia in the SHARE-HCAP subsample based on diagnostic criteria and Estimation approach^a^.

		Classified using Manly et al.^24^			Predicted^*b*^ using Hurd et al.^29^		
	Total sample, No.	Normal % (SE)	MCI % (SE)	Demented % (SE)	Normal % (SE)	MCI % (SE)	Demented % (SE)
Germany	547	76.9	18.8	4.3	77.6	17.6	4.8
		(1.8)	(1.7)	(0.9)	(1.8)	(1.6)	(0.9)
Italy	537	65.6	22.6	11.8	58.5	29.7	11.8
		(2.0)	(1.8)	(1.4)	(2.1)	(2.0)	(1.4)
France	528	71.8	22	6.2	72.2	21.2	6.6
		(2.0)	(1.8)	(1.0)	(1.9)	(1.8)	(1.1)
Denmark	573	77.1	18	4.9	76.1	19.1	4.8
		(1.8)	(1.6)	(0.9)	(1.8)	(1.6)	(0.9)
Czech Republic	502	71.5	20.4	8.1	73.1	19.7	7.2
		(2.0)	(1.8)	(1.2)	(2.0)	(1.8)	(1.2)
SHARE-HCAP subsample	2,687	72.6	20.4	7.0	71.5	21.5	7.0
		(0.9)	(0.8)	(0.5)	(0.9)	(0.8)	(0.5)

SE, standard error.

^a^ Classification and estimation of prevalence are based on weighted data.

^b^ Prevalence estimates generated from estimation equation, see Supplemental section S2.

### Probability of cognitive status in the SHARE parent sample

In assessing the cognitive status in the SHARE parent study, we distinguished between respondents who were able to complete the cognition items in Wave 9 (95.7%), those for whom health information was obtained by proxies (3.1%), and respondents who were excluded due to missing data (1.2%). For the first group, we used the regression equation developed in Step (c) to predict the probabilities of each individual being normal, MCI and demented, based on the same set of demographic, cognition and health variables in the SHARE parent sample. We recognize the uncertainty in classification by predicting probabilities rather than a cognitive class. The prevalence rates of normal, MCI and demented are then calculated as the country-specific average probabilities of each category.

Finally, we added the informants’ assessments of the cognitive status for the 3.1% of respondents who were not able to answer the cognition items in Wave 9, using a simple approach that was based on the informant’s assessment of the respondent’s memory function. If the respondent’s memory function was assessed poor (fair), the respondent was classified as demented (MCI), else normal. Details are provided in Supplementary Section S3 and Supplementary Table S6. All statistical analyses were conducted with Stata (version 14.2) and Mplus (version 8.10).

## Results

Table 3 shows the main result of this study: the estimated prevalence rates of MCI and dementia based on the cognition measures in Wave 9 of SHARE validated by the SHARE-HCAP results.

**Table 3:** Prevalence estimates for 27 European countries and Israel, using prediction model based on SHARE-HCAP and using cutoff based classification based on Langa-Weir method.

		HCAP-validated^*a*^				Langa-Weir^*b*^			
Country	N	MCI, % (SE)		Demented, % (SE)		MCI, % (SE)		Demented, % (SE)	
Austria	2,176	16.9	(0.8)	6.8	(0.5)	9.7	(0.6)	5.0	(0.5)
Germany	2,708	16.8	(0.7)	5.3	(0.4)	11.4	(0.6)	3.3	(0.3)
Sweden	2,010	17.2	(0.8)	5.0	(0.5)	10.9	(0.7)	2.7	(0.4)
Netherlands	1,686	20.5	(1.0)	5.7	(0.5)	11.8	(0.8)	2.1	(0.3)
Spain	1,458	29.1	(1.2)	22.7	(1.1)	28.7	(1.2)	29.1	(1.2)
Italy	2,761	24.9	(0.8)	11.6	(0.6)	21.7	(0.8)	14.6	(0.7)
France	2,035	19.9	(0.9)	6.0	(0.5)	13.9	(0.8)	5.8	(0.5)
Denmark	1,523	18.0	(1.0)	5.3	(0.6)	9.8	(0.8)	2.4	(0.4)
Greece	2,351	30.4	(0.9)	14.0	(0.7)	21.0	(0.8)	11.0	(0.6)
Switzerland	1,425	17.8	(1.0)	4.6	(0.5)	11.1	(0.8)	2.1	(0.4)
Belgium	2,783	21.1	(0.8)	8.3	(0.5)	14.1	(0.7)	5.4	(0.4)
Israel	660	24.7	(1.7)	19.5	(1.5)	17.2	(1.5)	15.3	(1.4)
Czech Republic	2,647	18.6	(0.7)	5.9	(0.4)	9.8	(0.6)	3.3	(0.3)
Poland	3,137	27.3	(0.8)	14.0	(0.6)	23.0	(0.7)	12.3	(0.6)
Luxembourg	546	19.2	(1.6)	7.2	(1.1)	10.8	(1.3)	6.4	(1.0)
Hungary	1,229	23.5	(1.2)	8.7	(0.8)	9.6	(0.8)	3.2	(0.5)
Portugal	924	31.1	(1.5)	21.1	(1.3)	32.0	(1.5)	28.6	(1.5)
Slovenia	2,772	23.3	(0.8)	11.1	(0.6)	19.6	(0.7)	8.9	(0.5)
Estonia	2,950	20.1	(0.7)	8.9	(0.5)	15.7	(0.7)	6.7	(0.5)
Croatia	2,858	26.8	(0.8)	14.6	(0.7)	21.7	(0.8)	13.6	(0.6)
Lithuania	921	26.4	(1.5)	13.9	(1.1)	23.0	(1.4)	13.5	(1.1)
Bulgaria	573	29.9	(1.9)	12.2	(1.4)	16.2	(1.5)	8.7	(1.2)
Cyprus	555	30.3	(2.0)	15.6	(1.6)	21.0	(1.7)	8.7	(1.2)
Finland	1,237	20.7	(1.1)	6.7	(0.7)	16.7	(1.0)	3.8	(0.5)
Latvia	1,026	27.0	(1.4)	10.1	(1.0)	22.1	(1.3)	12.3	(1.0)
Malta	654	29.3	(1.8)	12.2	(1.3)	24.6	(1.7)	11.8	(1.3)
Romania	994	28.5	(1.4)	16.7	(1.2)	25.1	(1.4)	16.1	(1.2)
Slovakia	591	28.7	(1.9)	11.2	(1.3)	25.3	(1.8)	7.7	(1.1)
**SHARE Wave 9**	**47**,**193**	**23.9**	(0.2)	**10.9**	(0.1)	**17.8**	(0.2)	**9.4**	(0.1)
**Coefficient of variation**				**0.46**				**0.80**	

SE, standard error

^a^ Prevalence estimates generated from estimation equation using the Hurd et al. approach.

^b^ Prevalence estimates generated from the Langa-Weir summary score. Originally, Langa-Weir does not classify MCI but rather cognitive impairment without dementia (CIND) which overlap conceptually as intermediate stages of cognitive health.

The cross-national variation in Europe is very large. The probability of being demented among individuals aged 65 and older ranges from around 5% in Switzerland, Sweden, Denmark and Germany to more than 20% in Spain and Portugal. On average across all 28 SHARE countries, it is 11% (SE = 0.1), roughly comparable to the results by Manly et al.^24^ for the US. Prevalence rates are particularly high in the Mediterranean and Southeastern countries, much higher than previously reported^1,3–9,13^.

MCI is on average 24% (SE = 0.2) in the 27 European countries and Israel, again varying greatly between Austria, Germany, Sweden, Denmark and Switzerland on the lower side (about 17%) and the Mediterranean and Southeastern countries on the higher side, reaching almost a third in Bulgaria, Cyprus, Greece, and Portugal.

Table 3 also compares our HCAP-validated prevalence estimates with estimates based on the original Langa-Weir (LW) scale^14^ that adds immediate and delayed word recall (0–20), serial 7s (0–5) and backwards counting (0–2). Demented is defined as (0–6) and normal as (12–27). Langa-Weir call the range in between (7–11) “CIND” (cognitively impaired but not demented) which compares to the MCI classification in^24^. The original LW scale has been validated against diagnostic information from the ADAMS study^30^. It deviates from the LW variant used in^10^ since backwards counting was not available in the 2017 SHARE data. The prevalence estimates based on the original LW scale are generally lower than the HCAP-validated estimates but exhibit a much larger variation as indicated by the coefficient of variation (i.e., the variance of the estimated effect scaled by the average effect size), with very low estimates e.g. in Switzerland and the Netherlands and much higher prevalence estimates e.g. in Spain and Portugal. We contribute the differences between the HCAP-validated and the original LW scales to the larger breadth of cognition measures in the HCAP-validated scale relative to the LW scale, reducing the impact of each single measure and thus providing a more robust measure of cognition. Moreover, the HCAP-validated results are validated against the in-depth assessment of the European SHARE-HCAP subsample while the LW scale in Table 3 is based on the thresholds derived from the US ADAMS study which may not represent Europe well. The MCI prevalence rates measured by the HCAP-validated scale (23.9% averaged over all countries) are much higher than measured by the LW scale (17.8%). We attribute this to the classification algorithm^24^ which includes additional perspectives on an individual’s cognitive function, e.g. informant reports and self-reports. These additional reports allow us to detect earlier and milder forms of cognitive impairment than the LW scale.

Table 4 shows that prevalence rates vary plausibly by age and education. The right-most panel shows the p-values of t-tests that compare each group (row) with the adjacent group (row below). Every 5-year increase in age increases the risk of dementia (all p-values below 0.0001). Women have an age-adjusted higher risk of MCI compared to men (*p* < 0.0001) but there is no significant difference in dementia. An important finding is the strong association on the international level between cognitive performance and the respondents’ educational achievement when they were young. An increase in the age and sex-adjusted level of education is associated with a decrease in the risk of both MCI and dementia (all p-values below 0.0001). The sex difference in MCI might reflect sex differences in education, possibly as a result of differences in access to education over and above what is measured by education^31^.

**Table 4:** Group differences in cognitive performance by age, sex and education.

Group			*Prevalence estimate*,* % (SE)*			*p-value of group differences* ^*a*^		
		Total sample, No.	Normal	MCI	Demented	Normal	MCI	Demented
Age, y	65–69	12,528	75.1	19.8	5.1			
			(0.2)	(0.2)	(0.1)	0.000	0.000	0.000
	70–74	12,567	71.4	22.3	6.3			
			(0.3)	(0.2)	(0.1)	0.000	0.001	0.000
	75–79	9,839	69.2	23.1	7.7			
			(0.3)	(0.2)	(0.2)	0.000	0.000	0.000
	80–84	7,028	63.4	25.6	11.0			
			(0.4)	(0.2)	(0.3)	0.000	0.000	0.000
	85–89	3,881	52.9	29.8	17.4			
			(0.6)	(0.3)	(0.5)	0.000	0.288	0.000
	90+	1,890	44.2	30.2	25.6			
			(0.9)	(0.5)	(0.9)			
Sex^b^	Female	27,015	69.3	22.1	8.6			
			(0.2)	0(0.1)	(0.1)	0.000	0.000	0.153
	Male	20,718	66.0	25.1	9.0			
			(0.2)	(0.1)	(0.1)			
Education^c^	≤ Primary school	8,745	58.2	28.1	13.7			
			(0.4)	(0.2)	(0.3)	0.000	0.000	0.000
	Some high school	8,298	64.0	25.7	10.3			
			(0.4)	(0.2)	(0.2)	0.000	0.000	0.000
	High school or some college	19,715	71.5	21.7	6.8			
			(0.2)	(0.1)	(0.1)	0.000	0.000	0.000
	≥ College degree	10,852	74.7	19.7	5.6			
			(0.3)	(0.2)	(0.1)			

^a^ p-values represent the results of pairwise difference tests between two consecutive groups within each state of cognition (normal, MCI, demented). For example, the first row of p-values is the pairwise difference test between the 70-74y group and 65-69y group.

^b^Age adjusted

^c^Age and sex adjusted

**Fig. 1:**
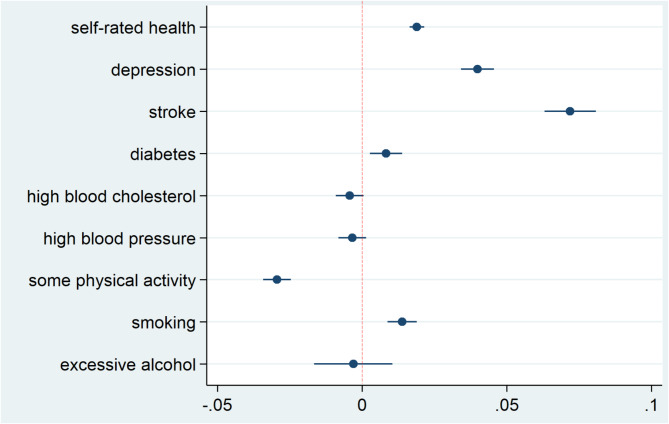
Association between the probability of being demented and frequent comorbidities of dementia. The blue dots show the percentage by which the presence of a comorbidity decreases/increases the probability of being demented. The error bands denote 95% confidence intervals. For example, having had a diagnose of a stroke increases the probability of being demented by 7.2% which is statistically significant, while high blood pressure does not have a statistically significant association with dementia conditional on the other co-morbidities. All associations are conditional on age, sex and education.

The probability of being demented also varies plausibly with comorbidities associated with dementia^21^ such as depression, stroke and diabetes, which gives our results additional face validity. Figure 1 shows the percentage by which the presence of a comorbidity decreases or increases the probability of being demented, obtained by a multivariate regression, which links the probability of dementia with a self-reported doctor’s diagnosis of the indicated conditions in Waves 1 through 9 of SHARE, conditional on the level of age, sex and education (Supplementary Table S7). A diagnosis of stroke increases the probability of being demented by 7.2% points, diabetes by 0.8% points. Depression is measured by the EURO-D scale^32^. A value exceeding 4 increases the probability of being demented by 4.0% points. These associations are statistically significant, while high blood pressure and high cholesterol values do not have a statistically significant association with dementia conditional on the other co-morbidities. All associations in Fig. 1 are conditional on age, sex and education.

Figure 1 also depicts associations with lifestyles that have been identified as risk factors for dementia^21^. Current physical activity reduces the probability of being demented while smoking increases it. We do not find a statistically significant association with excessive alcohol use in the current Wave 9.

**Fig. 2:**
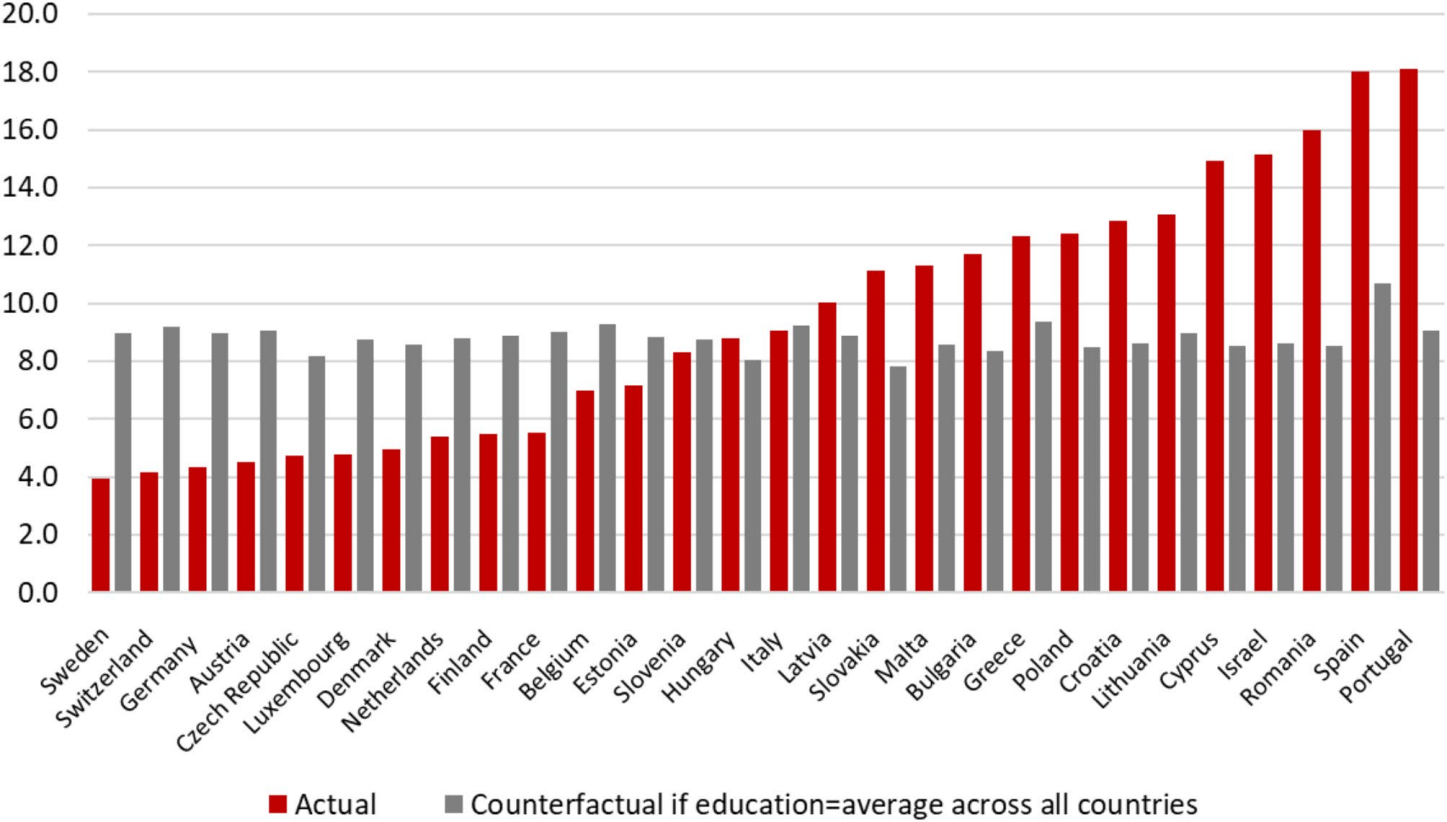
Prevalence of dementia for 27 European countries and Israel. Actual and counterfactual if education had been equal across all countries. The red bars show the actual estimated share of demented individuals in each country. The grey bars show the counterfactual share of demented individuals if education in each country had been equal to the average of the 28 countries.

Finally, an analogous regression approach can be used to shed light on the potential socioeconomic drivers of dementia and its disparities across Europe. Figure 2 shows how the probability of dementia would vary counterfactually across countries if education had been the same in all SHARE countries, namely the average of the 27 European countries and Israel. This variation is dramatically smaller than the actual variation, showing the strength of the association between education and the probability of being demented.

## Discussion

This paper provides up-to-date strictly cross-nationally comparable estimates of prevalence of mild cognitive impairment and dementia in 27 European countries and Israel, represented by a large sample of over 47,000 individuals aged 65 and older. As opposed to earlier European studies, these prevalence estimates are validated by a cognitive assessment using a globally harmonized protocol (HCAP) and a classification algorithm that is adopted by similar aging studies across the globe. Using HCAP as a validation base is an important methodological innovation and a significant step forward since it makes our prevalence estimates comparable to those of epidemiological and clinical studies, which have been the source for earlier international studies on dementia prevalence.

Our main finding is the large variation in the prevalence of MCI and dementia across Europe and Israel. Relative to earlier estimates, dementia prevalence is much higher in the Mediterranean and Eastern European countries. This in itself is an important finding for the entire field of dementia epidemiology, for both research and medical practice. It is important because it seems that the risk of dementia is seriously underestimated in many European countries with potential adverse consequences for healthcare planning and prevention.

Much of this variation can be explained by the large international differences in education, reflecting the differences in national education systems when the SHARE respondents were young. This is another important result which has implications far beyond Europe. It may explain the disproportionate burden of dementia and MCI among African Americans in the US as well as the global differences in dementia reported by GBD and OECD. This finding is also a potential anchor for preventative measures^21^. Sex differences in education may also explain the difference in MCI prevalence between men and women^31^, especially in the South of Europe women had less access to education.

The extent to which the association between education and cognition is causal is a matter of controversy and interpretation, since education in early life puts individuals on different occupational, economic and lifestyle paths during mid and late life which in turn have their own causal effects on cognition^33–35^. Moreover, further work is needed to consider the cross-national variation in other risk factors such as those mentioned in the 2024 Lancet report^21^.

The study rests on a set of critical assumptions. First, we assume that the five SHARE-HCAP countries are sufficiently representative to act as validation for the European context and provide weights for the cognition items that apply for all of Europe and Israel. Since there is substantial inhomogeneity within these five countries, even more inhomogeneity may be expected for all 27 European countries and Israel. Future work thus needs to extend the number of countries covered by HCAP assessment.

Similarly, a second assumption is that the Manly et al.^24^ thresholds immanent to the HCAP classification algorithm apply to all SHARE countries equally. Without a “gold standard” calibration target for the European countries and Israel such as the US ADAMS study^30^, we prefer this approach to relying on estimates that suffer from the shortcomings described in the introduction.

A third critical assumption is the validity of the regression-based refinement of the cognition indicators in Waves 8 and 9 with the help of the SHARE-HCAP classification results. Validity requires a sufficient accuracy of the prediction equation and a reasonable extent of consistency between the cognition measurements in SHARE-HCAP and the SHARE parent study. We believe that Table 2 documents this validity.

Cognition measures in observational studies are noisy, exhibit substantial test-retest variation and often fail to correspond with respondent-reported doctor diagnoses. This noisiness limits the precision of the probability estimates for each individual but much less so for the country-specific prevalence rates due to the large sample size of the SHARE parent sample. This is indicated by the standard errors in Table 3.

Finally, our results may underestimate the prevalence of dementia because non-response tends to be higher for individuals with dementia. We have spent much effort to minimize such bias, most importantly by assessing the individuals’ cognitive performance with the help of proxies (family members or friends) and by following individuals when they move into a nursing home or similar institutions where proxies include nurses.

## Electronic supplementary material

Below is the link to the electronic supplementary material.

Supplementary Material 1

## Data Availability

Data for the Survey of Health, Ageing and Retirement in Europa is available for the scientific community at https://share-eric.eu/data/.
