# Supplementary material for: Harmonized prevalence estimates of dementia in Europe vary strongly with childhood education
Source: Sci Rep. 2025 Apr 23;15:14024. doi: 10.1038/s41598-025-97691-z (PMC12019132; doi:10.1038/s41598-025-97691-z)
Supplement: Supplementary file 1 — Supplementary Material 1 [file 41598_2025_97691_MOESM1_ESM.docx]

**Supplementary Information**

**Harmonized Prevalence Estimates of Dementia in Europe
Vary Strongly with Childhood Education**

**Axel Börsch-Supan, PhD* ^1,2,3^, Salima Douhou, PhD^1,2^,
Marcela C. Otero, PhD^1,2^, Beatrice Baaba Tawiah, PhD^2^**

**^1^Max Planck Institute for Social Law and Social Policy, Munich, Germany
^2^Munich Research Institute for the Economics of Aging and SHARE Analyses, Munich, Germany
^3^National Bureau of Economic Research, Cambridge, Massachusetts, USA**

**02 April 2025**

**Table of contents**

[**S1. Classification of cognitive status in the SHARE-HCAP sample** 2](#_Toc188880746)

[**S2. Regression approach to predict prevalence rates in the SHARE parent sample** 3](#_Toc188880747)

[**S3. Classification of respondents who were unable to do the cognition tests** 6](#_Toc188880748)

[**Table S1. Sample characteristics of SHARE Wave 9 and SHARE-HCAP subsample, weighted**^a^ 7](#_Toc188880749)

[**Table S2: Share and cognitive performance of proxy respondents (number and percentages)** 8](#_Toc188880750)

[**Table S3. Cognitive tests and informant items** 9](#_Toc188880751)

[**Table S4. Description of cognitive variables** 10](#_Toc188880752)

[**Table S5. Ordered probit regression in SHARE-HCAP subsample** 11](#_Toc188880753)

[**Table S6. Prevalence estimates based on regressions with and without Wave 8 data (percent, standard errors in parentheses)** 12](#_Toc188880754)

[**Table S7. Regression of probability of being demented on comorbidities, age, sex and education** 14](#_Toc188880755)

[**References** 15](#_Toc188880756)

**S1. Classification of cognitive status in the SHARE-HCAP sample**

For the classification into normal, MCI or dementia in the SHARE-HCAP sample we followed the three-stage approach that has been described in Manly et al.[1].

First, we selected a normative sample from the SHARE-HCAP sample. The exclusion criteria were based on conditions related to pathological cognitive ageing. Specifically, individuals with neurodegenerative disease, stroke or significant cognitive or functional impairment were excluded from the normative sample. The resulting normative sample included 1,605 individuals from the SHARE-HCAP sample.

Second, we used factor analysis to derive factor score estimates in five domains of cognition: memory, executive functioning, orientation, visuospatial and language. The factor score estimates within the normative sample were rank normalized and then adjusted for age, gender, education and country of residence.

Third, classification of dementia required that at least two cognitive domains were 1.5 SDs below the mean of the normative sample and functional impairment reported by an informant. Individuals who did not meet the criteria for cognitive impairment in any domain were classified as normal. If one cognitive domain was in the impaired range, individuals were classified as normal only if one cognitive domain score was below 1.5 SDs and no informant report on their cognitive functioning and they did not self-report cognitive concerns. All other participants were classified as MCI.

**S2. Regression approach to predict prevalence rates in the SHARE parent sample**

Our preferred classification is based on diagnostic criteria and follows the approach by Manly et al for the SHARE-HCAP study. We then employ the regression-based approach from Hurd et al.[2] to mimic this classification in the full SHARE parent study. More specifically, we predict the probability of being normal, MCI or dementia rather than relying on a single cognitive measure or other types of summary scores that have been used in the past and base this prediction on the cognition measures in the full SHARE parent study, weighing these measures exactly as in the SHARE-HCAP study.

In a first step, using the SHARE-HCAP sample, we employ an ordered probit model[3] to relate the outcome of the classification (normal, MCI, dementia) to a selection of demographic variables and cognitive and health measures that is drawn from existing research and are available both in the SHARE-HCAP subsample and the SHARE parent study. Ordered probit models can be used to examine how covariates are related to a categorical outcome variable, where the ordering of the categories of the outcome variable has real world interpretation. In the current study, the outcome variable is a three-category measure of cognitive status, which takes on values of 1, 2, and 3 for individuals assessed as normal, MCI and demented, respectively. Note that the actual values of the outcome (here: 1, 2 or 3) are irrelevant, only their ordering matters.

An ordered probit model posits that the values of the categorical outcome variable are determined by an unobserved index variable, y*, which in our case can be thought of as cognitive functioning. Individuals with y* below some cutoff, c1, are classified as normal, those with y* above some cutoff, c2, are classified as demented, and those with y* in between c1 and c2 are classified as MCI. The ordered probit model further assumes that the unobserved index variable y* is a linear function of the right-hand-side variables plus a random term that is distributed standard-normal. The coefficients of the explanatory variables and the cutoffs c1 and c2 are estimated via maximum likelihood.

More formally, the regression equation is:

**(1) Prob(cogclass_t_ = i) = OPROB(age_t_, sex_t_, educ_t_, country_t_, cogn_t-1_, health_t-1_, Δcogn_t-1_, Δhealth_t-1_),**

where cogclass denotes the classification obtained by the Manly et al. approach in the SHARE-HCAP sample, *t* the time of the SHARE-HCAP data collection, *t-1* Wave 9 about five months earlier to SHARE-HCAP (in 2022), and Δx*_t-1_* the change of variable x between Wave 9 and Wave 8, two years earlier.

Age is measured as 5-year age bands, and education by ISCED1997. [4] A set of country dummies is included and scaled such that their average is exactly zero. This reflects the fact that country-specific effects are unknown in the 23 countries that are not represented in the SHARE-HCAP sample.

Cognition measures included are orientation in time, immediate and delayed word recall, serial 7s, and animal naming, see Table S4.

As health measures, we selected the sum of activities of daily living (ADL) and the sum of instrumental activities of daily living (IADL). The cognition and health measures refer to Wave 9 and Wave 8 to avoid circularity issues with the dependent variables that were computed using the SHARE-HCAP data. We distinguish three groups of respondents. (a) For respondents, who participated in Wave 8 and Wave 9 and were able to answer the relevant questions by themselves, we used Equation 1. (b) For respondents, who participated in Wave 9 and were able to answer the relevant questions by themselves in Wave 9 but did not participate in Wave 8 where fieldwork had to be cut short due to the COVID-19 pandemic, we used Equation 1 without Δcogn_t-1_ and Δhealth_t-1_. (c) Respondents in Wave 9 who were unable to complete the cognitive measures are not included in the regression analysis but the results of their informant reports were included in our prevalence estimates, see Section S3. Together, these three groups cover 47,193 of the 47,733 observations in Wave 9, i.e., the analytic sample covers 98.9% of the total sample. The remaining 1.1% includes respondents with missing information about education and selected health items. Since this proportion is very small, we did not impute these observations. The regressions were weighted to take into account potential heteroscedasticity due to potential differences in measurement error e.g. by country, age and education.

Table S5 shows the regression results for those respondents who participated in both Wave 8 and 9 (N=1,909). The prevalence prediction results excluding Δcogn_t-1_ and Δhealth_t-1_ are very similar to (a) as documented in Table S6. Pseudo R-squared is 23.3%. We report robust standard errors to account for remaining heteroscedasticity after weighting. Coefficients for age and education show the expected pattern. There is no significant difference between men and women. The country dummies reflect the country-specific prevalence rates. The presence of ADLs and IADLs increase the probability of cognitive impairment. Immediate and delayed word recall, orientation to month and animal naming are the best predictors for cognitive impairment in terms of statistical significance, similarly but weaker for the change in these measures between Waves 8 and 9.

The estimated regression equation was then used to predict Prob(cogclass_t_ = i) in the full SHARE parent sample of all 28 countries, i.e., we replaced the right-hand-side variables in equation (1) by their equivalents in the full SHARE parent sample to obtain the probabilities for normal, MCI and dementia on the left-hand-side of equation (1). This means that the primary input for the “HCAP-validated prevalence rates” are the cognition measures in Wave 9, aggregated into a scale that has the same weights for each cognition measure as in the SHARE-HCAP study.

**S3.** **Classification of respondents who were unable to do the cognition tests**

1,479 individuals in the SHARE parent study, 3.1% of the total sample, were unable to do the cognition tests in Wave 9. For these individuals, we asked a member of the family or a friend (“proxy” or “informant”) to give short report on the cognitive status of the individual. They assessed their memory ability from excellent to poor and stated whether a respondent could not be left alone, gets lost, wanders off, or hears or sees things that do not exist.

Table S2 shows the share of proxy interviews by country, ranging from 0.5% in Slovakia to 9.9% in Portugal. Of these 1,479 individuals, 42 individuals were also selected to participate in SHARE-HCAP. Since this overlap is too small to do a regression analysis similar to Equation 1, we took a less involved approach and classified these individuals according to the answers given by the informant. We classified respondents as demented if their memory was assessed poor or if the informant stated that the respondent could not be left alone. We classified respondents as MCI if their memory was assessed fair, if the informant stated that the respondent tends to get lost, wanders off, or hears or sees things that do not exist. All others were classified as normal. Table S2 shows the resulting distribution of the proxy respondents by so assessed cognitive performance. Most of the proxy respondents were assessed as having dementia (61.0%) or MCI (20.2%) but a considerable share (18.8%) was assessed normal and could not be interviewed in the main study due to reasons unrelated to their cognitive performance, e.g., due to a temporary illness or prolonged absence from their usual place of residence. In the small overlap between SHARE-HCAP and Wave 9 proxy interviews (N=42), 69% were classified demented, 23% MCI and 4% normal using the SHARE-HCAP criteria reported in Section 2b of the main text.

**Table S1. Sample characteristics of SHARE Wave 9 and SHARE-HCAP subsample, weighted**^a^

| **Country** | **Total Sample, No.** | **Res-ponse rate^b^, %** | **Age, mean (SD), years** | | **Female %** | **Male %** | **≤ primary school^c^, %** | **Some high school^c^, %** | **High school or some college %** | **≥ college degree %** | **Health: ADL+IADL^d^, mean (SD)** | | **HH income^e^, median in Euro (IQR)** | | |
| --- | --- | --- | --- | --- | --- | --- | --- | --- | --- | --- | --- | --- | --- | --- | --- |
| Germany | 547 | 76.1 | 75.5 | (7.2) | 55.9 | 44.1 | 0.3 | 10.8 | 52.5 | 36.4 | 1.0 | (2.2) | 2300 | (1600) | |
| Italy | 537 | 79.4 | 75.8 | (7.5) | 56.1 | 43.9 | 42.9 | 26.8 | 24.1 | 6.3 | 0.9 | (2.2) | 1400 | (1350) | |
| France | 528 | 74.5 | 75.3 | (7.7) | 56.6 | 43.4 | 25.4 | 7.5 | 37.2 | 29.9 | 0.9 | (1.9) | 2200 | (1800) | |
| Denmark | 573 | 76.3 | 75.1 | (7.3) | 53.9 | 46.1 | 9.4 | 10.0 | 35.1 | 45.6 | 0.6 | (1.5) | 2554 | (2110) | |
| Czech Republic | 502 | 72.6 | 74.4 | (6.9) | 56.7 | 43.3 | 7.1 | 22.8 | 54.6 | 15.4 | 1.0 | (2.3) | 773 | (571) | |
| **SHARE-HCAP subsample** | **2687** | **75.8** | **75.5** | **(7.5)** | **56.2** | **43.8** | **20.0** | **14.9** | **39.8** | **25.3** | **0.9** | **(2.1)** | **2000** | **(1700)** | |
| Austria | 2204 | 60.8 | 75.7 | (7.5) | 55.8 | 44.2 | 9.8 | 12.0 | 49.4 | 28.8 | 1.1 | (2.7) | 2200 | (1600) | |
| Germany | 2750 | 70.7 | 75.8 | (7.5) | 55.4 | 44.6 | 1.1 | 11.3 | 53.9 | 33.7 | 1.0 | (2.4) | 2400 | (1700) | |
| Sweden | 2054 | 58 | 75.1 | (7.4) | 55.3 | 44.7 | 15.3 | 14.4 | 35.4 | 34.9 | 0.7 | (2.1) | 2258 | (1878) | |
| Netherlands | 1760 | 48.9 | 74.9 | (7.3) | 54.7 | 45.3 | 7.5 | 33.5 | 27.4 | 31.6 | 0.7 | (1.9) | 2400 | (1650) | |
| Spain | 1433 | 59 | 78.4 | (8.0) | 56.3 | 43.7 | 60.8 | 21.0 | 8.5 | 9.7 | 2.1 | (4.1) | 1200 | (1010) | |
| Italy | 2825 | 75.1 | 76.3 | (7.7) | 56.8 | 43.2 | 44.3 | 26.6 | 21.9 | 7.2 | 1.3 | (3.1) | 1400 | (1000) | |
| France | 2068 | 50.4 | 75.8 | (7.9) | 55.7 | 44.3 | 27.6 | 8.5 | 35.4 | 28.5 | 0.9 | (2.3) | 2300 | (1900) | |
| Denmark | 1544 | 60.6 | 75.0 | (7.3) | 54.3 | 45.7 | 8.2 | 7.9 | 37.5 | 46.4 | 0.7 | (1.9) | 2688 | (2231) | |
| Greece | 2360 | 67.5 | 76.0 | (7.6) | 54.8 | 45.2 | 46.2 | 10.8 | 25.0 | 18.1 | 1.3 | (2.8) | 850 | (600) | |
| Switzerland | 1433 | 70.2 | 75.4 | (7.8) | 55.1 | 44.9 | 9.0 | 10.6 | 61.4 | 19.0 | 0.5 | (1.6) | 3981 | (3683) | |
| Belgium | 2813 | 64.1 | 75.7 | (8.0) | 53.7 | 46.3 | 15.2 | 22.0 | 26.3 | 36.6 | 1.3 | (2.8) | 2200 | (1600) | |
| Israel | 666 | 24.9 | 73.7 | (7.1) | 56.1 | 43.9 | 22.4 | 12.3 | 26.2 | 39.1 | 1.6 | (3.5) | 2829 | (2942) | |
| Czech Republic | 2674 | 67.8 | 73.9 | (6.8) | 58.4 | 41.6 | 7.1 | 22.8 | 52.9 | 17.2 | 0.9 | (2.4) | 977 | (733) | |
| Poland | 3165 | 79.1 | 74.1 | (7.6) | 60.0 | 40.0 | 14.7 | 18.4 | 55.2 | 11.7 | 1.3 | (3.1) | 640 | (576) | |
| Luxembourg | 589 | 50.3 | 74.8 | (7.5) | 52.7 | 47.3 | 27.8 | 12.1 | 37.1 | 22.9 | 0.8 | (2.4) | 4000 | (3300) | |
| Hungary | 1234 | 58.1 | 73.6 | (6.8) | 61.7 | 38.3 | 0.6 | 29.1 | 57.4 | 12.9 | 1.1 | (2.3) | 419 | (332) | |
| Portugal | 933 | 64.4 | 75.9 | (7.2) | 60.6 | 39.4 | 67.1 | 9.5 | 9.8 | 13.6 | 2.0 | (4.0) | 850 | (800) | |
| Slovenia | 2805 | 76.2 | 74.9 | (7.7) | 56.4 | 43.6 | 8.1 | 24.5 | 51.4 | 16.0 | 1.3 | (3.2) | 1200 | (1070) | |
| Estonia | 2984 | 77.3 | 75.3 | (7.6) | 64.9 | 35.1 | 2.6 | 21.4 | 52.3 | 23.8 | 1.2 | (2.7) | 620 | (581) | |
| Croatia | 2856 | 82.8 | 74.8 | (7.3) | 58.7 | 41.3 | 20.7 | 20.0 | 44.1 | 15.3 | 1.4 | (3.2) | 531 | (597) | |
| Lithuania | 923 | 76.6 | 75.7 | (8.0) | 66.7 | 33.3 | 10.0 | 11.9 | 41.5 | 36.7 | 1.5 | (3.3) | 650 | (590) | |
| Bulgaria | 575 | 75.4 | 74.4 | (6.8) | 60.1 | 39.9 | 9.6 | 28.6 | 49.6 | 12.2 | 1.2 | (2.5) | 276 | (253) | |
| Cyprus | 553 | 63.7 | 74.5 | (7.2) | 55.4 | 44.6 | 48.4 | 10.0 | 25.6 | 16.0 | 1.2 | (3.2) | 4500 | (14000) | |
| Finland | 1264 | 63.9 | 75.0 | (7.3) | 54.8 | 45.2 | 24.0 | 6.9 | 30.3 | 38.8 | 0.7 | (1.9) | 2200 | (2000) | |
| Latvia | 1031 | 80.1 | 75.4 | (7.3) | 65.8 | 34.2 | 4.8 | 13.8 | 55.3 | 26.0 | 0.9 | (2.1) | 470 | (462) | |
| Malta | 654 | 75.7 | 74.4 | (7.3) | 54.4 | 45.6 | 55.9 | 0.7 | 37.0 | 6.4 | 0.8 | (2.5) | 1150 | (1500) | |
| Romania | 990 | 91.2 | 74.3 | (7.6) | 55.9 | 44.1 | 20.0 | 37.9 | 37.9 | 4.2 | 1.5 | (3.4) | 385 | (416) | |
| Slovakia | 593 | 88.9 | 73.5 | (6.8) | 56.7 | 43.3 | 2.7 | 15.8 | 76.5 | 5.0 | 1.0 | (2.5) | 800 | (580) | |
| **SHARE parent  Wave 9** | **47,733** | **68.4** | **75.6** | **(7.7)** | **56.6** | **43.4** | 23.2 | 17.7 | 37.7 | 21.4 | **1.2** | **(2.9)** | **1600** | **(1800)** | |
| Abbreviations: ADL, Activities of Daily Living; IADL, Instrumental Activities of Daily Living; HH income, Household income | | | | | | | | | | | | | | |  |
| ^a^ Indicates sample characteristics using sampling weights. | | | | | | | | | | | | | | |  |
| ^b^ Response rates are the ratio of number of individuals that completed an interview to the number of individuals that were eligible for an interview in SHARE Wave 9. | | | | | | | | | | | | | | |  |
| ^c^  Educational attainment is measured using the International Standard Classification of Education (ISCED) 1997 ^4^: ≤ primary school (ISCED level 0 and 1), Some high school (ISCED level 2), High school or some college (ISCED level 3 and 4), ≥ college degree (ISCED level 5 and 6). | | | | | | | | | | | | | | |  |
| ^d^ Combined measure of limitations in ADLs and IADLs, which are measured using self-report and reflect functional status and independence. | | | | | | | | | | | | | | |  |
| ^e^ Total household income per month (average), expressed in euros. | | | | | | | | | | | | | | |  |

**Table S2: Share and cognitive performance of proxy respondents
(number and percentages)**

|  | **Number  of proxy** | **Share of proxy** |  | **Cognitive performance** | | |
| --- | --- | --- | --- | --- | --- | --- |
| **Country** | **interviews** | **interviews** |  | **Normal** | **MCI** | **Demented** |
| Austria | 111 | 5.0 |  | 37.8 | 19.8 | 42.3 |
| Germany | 45 | 1.6 |  | 13.3 | 24.4 | 62.2 |
| Sweden | 39 | 1.9 |  | 18.0 | 23.1 | 59.0 |
| Spain | 119 | 8.3 |  | 12.6 | 12.6 | 74.8 |
| Italy | 129 | 4.6 |  | 7.7 | 17.7 | 74.6 |
| Greece | 61 | 2.6 |  | 3.3 | 13.1 | 83.6 |
| Belgium | 67 | 2.4 |  | 17.9 | 19.4 | 62.7 |
| Israel | 59 | 8.9 |  | 15.3 | 17.0 | 67.8 |
| Czech Republic | 46 | 1.7 |  | 21.7 | 23.9 | 54.4 |
| Poland | 140 | 4.4 |  | 15.0 | 25.7 | 59.3 |
| Portugal | 92 | 9.9 |  | 30.4 | 21.7 | 47.8 |
| Slovenia | 167 | 6.0 |  | 15.6 | 30.5 | 53.9 |
| Estonia | 90 | 3.0 |  | 20.0 | 17.8 | 62.2 |
| Croatia | 130 | 4.6 |  | 36.2 | 13.1 | 50.8 |
| **All 28 countries** | **1,479** | **3.1** |  | **18.8** | **20.2** | **61.0** |

Countries with less than 30 observations not reported

**Table S3. Cognitive tests and informant items**

| **Respondent tests of SHARE-HCAP** |
| --- |
| Mini Mental State Examination (MMSE)[5,6] |
| HRS TICS (3 items: Object naming; naming president)[7] |
| CERAD Word List – Recall: Immediate and delayed, Recognition[8-10] |
| Semantic Fluency (Animal Naming)[8,11-13] |
| Symbol cancellation test[14] |
| Timed Backward Counting Task[15] |
| Brief Community Screening Instrument for Dementia (CSI-D; 4 items)[16] |
| Story recall – immediate, delayed and recognition[17,18] |
| CERAD Constructional Praxis – immediate and delayed[5,19] |
| Symbol Digit Modalities Test (SDMT)[20] |
| HRS Number Series[21] |
| Raven’s Standard Progressive Matrices[22-24] |
| Trail Making Test (Part A and Part B)[25,26] |
| **SHARE-HCAP informant report items** |
| Background information |
| Jorm Informant Questionnaire on Cognitive Decline in the Elderly (IQCODE)[27] |
| Blessed Dementia Rating Scale[28] |
| HRS Activities |
| 10/66 Dementia Research Group Informant Questionnaire (4 items)[29] |
| CSI-D Cognitive Activities[30] |

**Table S4. Description of cognitive variables**

| **Cognitive variables** | **Description** |
| --- | --- |
| Immediate recall | Ten words list learning first trial: Recall as many of the words as you can in any order. |
| Delayed recall | Ten words list learning delayed recall: A little while ago, I read you a list of words and you repeated the ones you could remember. Please tell me any of the words that you can remember now? |
| Serial 7 | Subtract 7 from 100, and continue subtracting 7 from each subsequent number for a total of five trials. |
| Orientation to day | “Which day of the month is it?” |
| Orientation to month | “Which month is it?” |
| Orientation to year | “Which year is it?” |
| Orientation to week | “Can you tell me what day of the week it is?” |
| Animal naming | Name as many animals as you can think of. |
| ADLs | Number of limitations with activities of daily living (ADL) reported |
| IADLs | Number of limitations with instrumental activities of daily living (IADL) reported |

**Table S5. Ordered probit regression in SHARE-HCAP subsample**

|  | **Coefficient** | **Robust Std. Error** |
| --- | --- | --- |
| Female | -0.164 | 0.103 |
| *Age* |  |  |
| 60-64 | *(ref)* |  |
| 65-69 | 0.398 | 0.089 |
| 70-74 | 0.243 | 0.059 |
| 75-79 | 0.248 | 0.058 |
| 80-84 | 0.159 | 0.039 |
| 85-89 | 0.159 | 0.037 |
| 90-94 | -0.226 | 0.073 |
| 95-99 | -0.847 | 0.529 |
| 100+ | -2.032 | 0.626 |
| Primary or less | *(ref)* |  |
| Some high school | 0.194 | 0.243 |
| High school or some college | 0.277 | 0.178 |
| College degree or higher | 0.550 | 0.192 |
| Germany | -0.162 | 0.120 |
| Italy | -0.166 | 0.180 |
| Denmark | 0.096 | 0.121 |
| Czech Republic | 0.185 | 0.133 |
| France | *(ref)* |  |
| ADLs in wave 9 | 0.127 | 0.089 |
| IADLs in wave 9 | 0.148 | 0.056 |
| Change in ADLs between waves 8 and 9 | -0.063 | 0.073 |
| Change in IADLs between waves 8 and 9 | -0.038 | 0.051 |
| *Test scores in wave 9* |  |  |
| Immediate recall | -0.104 | 0.057 |
| Delayed recall | -0.115 | 0.046 |
| Serial 7 | -0.098 | 0.045 |
| Orientation to day | 0.278 | 0.161 |
| Orientation to month | 1.319 | 0.519 |
| Orientation to year | 0.439 | 0.341 |
| Orientation to week | -0.482 | 0.349 |
| Animal naming | -0.069 | 0.015 |
| *Change in test scores between waves 8 and 9* |  |  |
| Immediate recall | 0.083 | 0.042 |
| Delayed recall | 0.025 | 0.037 |
| Serial 7 | 0.072 | 0.049 |
| Orientation to day | 0.100 | 0.123 |
| Orientation to month | -1.349 | 0.423 |
| Orientation to year | -0.037 | 0.255 |
| Orientation to week | 0.024 | 0.286 |
| Animal naming | 0.022 | 0.012 |
| N | 1,909 |  |

**Table S6. Prevalence estimates based on regressions with and without Wave 8 data (percent, standard errors in parentheses)**

|  | **Health and cognition  in Wave 9 only** | | |  | **Health and cognition  in Waves 8 and 9** | | |
| --- | --- | --- | --- | --- | --- | --- | --- |
| **Country** | **Normal** | **MCI** | **Demented** |  | **Normal** | **MCI** | **Demented** |
| Austria | 77.5 | 17.3 | 5.2 |  | 79.3 | 16.2 | 4.5 |
|  | (0.5) | (0.4) | (0.2) |  | (0.5) | (0.4) | (0.1) |
| Germany | 78.5 | 17.1 | 4.4 |  | 79.2 | 16.5 | 4.3 |
|  | (0.6) | (0.5) | (0.2) |  | (0.4) | (0.3) | (0.1) |
| Sweden | 78.7 | 17.4 | 3.9 |  | 79.0 | 17.1 | 3.9 |
|  | (0.9) | (0.8) | (0.2) |  | (0.4) | (0.4) | (0.1) |
| Netherlands | 74.2 | 20.3 | 5.5 |  | 74.3 | 20.3 | 5.4 |
|  | (0.8) | (0.7) | (0.2) |  | (0.6) | (0.5) | (0.2) |
| Spain | 51.1 | 30.9 | 18.0 |  | 51.7 | 30.3 | 18.0 |
|  | (1.1) | (0.9) | (0.6) |  | (0.9) | (0.8) | (0.5) |
| Italy | 68 | 23.9 | 8.0 |  | 64.4 | 26.6 | 9.0 |
|  | (0.6) | (0.5) | (0.2) |  | (0.6) | (0.5) | (0.2) |
| France | 74.2 | 20.4 | 5.4 |  | 74.6 | 19.9 | 5.5 |
|  | (0.9) | (0.8) | (0.2) |  | (0.5) | (0.4) | (0.1) |
| Denmark | 77.1 | 18.2 | 4.8 |  | 77.3 | 17.8 | 4.9 |
|  | (0.9) | (0.8) | (0.2) |  | (0.5) | (0.4) | (0.1) |
| Greece | 58.3 | 30.0 | 11.7 |  | 56.5 | 31.2 | 12.3 |
|  | (1.1) | (0.9) | (0.4) |  | (0.6) | (0.5) | (0.3) |
| Switzerland | 77.9 | 17.9 | 4.2 |  | 78.2 | 17.7 | 4.2 |
|  | (1.4) | (1.2) | (0.3) |  | (0.5) | (0.4) | (0.1) |
| Belgium | 71.7 | 21.3 | 7.0 |  | 71.9 | 21.1 | 7.0 |
|  | (0.5) | (0.4) | (0.2) |  | (0.6) | (0.5) | (0.2) |
| Israel | 60.8 | 25.6 | 13.5 |  | 59.4 | 25.4 | 15.1 |
|  | (1.9) | (1.5) | (0.9) |  | (1.1) | (0.9) | (0.6) |
| Czech Republic | 74.7 | 19.7 | 5.6 |  | 77.3 | 18.0 | 4.7 |
|  | (0.6) | (0.5) | (0.2) |  | (0.4) | (0.4) | (0.1) |
| Poland | 61.6 | 27.0 | 11.3 |  | 59.9 | 27.7 | 12.4 |
|  | (0.6) | (0.5) | (0.3) |  | (0.6) | (0.5) | (0.3) |
| Luxembourg | 76.5 | 18.6 | 4.9 |  | 76.1 | 19.1 | 4.8 |
|  | (1.9) | (1.6) | (0.5) |  | (0.9) | (0.7) | (0.2) |
| Hungary | 69.8 | 23.0 | 7.2 |  | 67.2 | 24 | 8.8 |
|  | (0.9) | (0.7) | (0.3) |  | (0.9) | (0.7) | (0.3) |
| Portugal | 49.8 | 32.1 | 18.1 |  | na | na | na |
|  | (0.9) | (0.8) | (0.5) |  | na | na | na |
| Slovenia | 68.4 | 23.1 | 8.5 |  | 69.0 | 22.7 | 8.3 |
|  | (0.7) | (0.6) | (0.3) |  | (0.5) | (0.4) | (0.2) |
| Estonia | 72.0 | 20.6 | 7.5 |  | 72.8 | 20.0 | 7.2 |
|  | (0.7) | (0.5) | (0.2) |  | (0.5) | (0.4) | (0.2) |
| Croatia | 59.6 | 27.5 | 12.9 |  | 59.9 | 27.3 | 12.8 |
|  | (0.6) | (0.5) | (0.3) |  | (0.7) | (0.6) | (0.3) |
| Lithuania | 62.5 | 25.5 | 12.0 |  | 60.0 | 27.0 | 13.1 |
|  | (2.1) | (1.7) | (0.9) |  | (0.9) | (0.7) | (0.4) |
| Bulgaria | 60.6 | 28.5 | 10.9 |  | 57.8 | 30.4 | 11.7 |
|  | (2.3) | (2.0) | (0.9) |  | (1.1) | (1.0) | (0.5) |
| Cyprus | 57.1 | 29.5 | 13.4 |  | 53.8 | 31.2 | 14.9 |
|  | (1.5) | (1.3) | (0.7) |  | (1.5) | (1.3) | (0.8) |
| Finland | 73.0 | 21.2 | 5.9 |  | 74.0 | 20.5 | 5.5 |
|  | (0.8) | (0.7) | (0.2) |  | (0.8) | (0.7) | (0.2) |
| Latvia | 64.9 | 26.1 | 9.0 |  | 62.1 | 27.8 | 10.0 |
|  | (1.2) | (1.0) | (0.4) |  | (0.9) | (0.8) | (0.4) |
| Malta | 61.8 | 28.1 | 10.1 |  | 59.1 | 29.6 | 11.3 |
|  | (1.8) | (1.5) | (0.7) |  | (1.1) | (1.0) | (0.5) |
| Romania | 56.8 | 28.6 | 14.6 |  | 55.1 | 28.9 | 16.0 |
|  | (1.5) | (1.3) | (0.8) |  | (0.9) | (0.8) | (0.5) |
| Slovakia | 61.7 | 28.2 | 10.1 |  | 59.9 | 29.0 | 11.1 |
|  | (2.3) | (2.0) | (0.9) |  | (1.1) | (0.9) | (0.4) |
| **Mean** | **67.1** | **23.8** | **9.1** |  | **66.4** | **24.1** | **9.5** |
|  | (0.2) | (0.1) | (0.1) |  | (0.1) | (0.1) | (0.1) |

Note: The left set of columns reports the predicted prevalences if the underlying regression is based on the subsamples of those respondents who were sampled in Wave 9 only; the left set of columns reports the predicted prevalences if the underlying regression is based on the subsamples of those respondents who were sampled in both Waves 8 and 9.

**Table S7. Regression of probability of being demented on comorbidities, age, sex and education**

|  | **Coefficient** | **Std. Err.** | **t-Stat** | **Prob>t** | **95% Confidence Interval** | |
| --- | --- | --- | --- | --- | --- | --- |
| Age | -0.039 | 0.0033 | -11.80 | 0 | -0.0449 | -0.0321 |
| Age^2^ | 0.000 | 0.0000 | 12.77 | 0 | 0.0002 | 0.0003 |
| Female | -0.031 | 0.0024 | -13.04 | 0 | -0.0353 | -0.0260 |
| Some high school | -0.011 | 0.0042 | -2.68 | 0.007 | -0.0195 | -0.0030 |
| High school or some college | -0.032 | 0.0035 | -9.10 | 0 | -0.0392 | -0.0253 |
| College degree or higher | -0.033 | 0.0038 | -8.63 | 0 | -0.0407 | -0.0256 |
| Self-rated health | 0.019 | 0.0013 | 14.67 | 0 | 0.0164 | 0.0215 |
| EURO-D | 0.040 | 0.0029 | 13.77 | 0 | 0.0342 | 0.0456 |
| Stroke | 0.072 | 0.0045 | 15.94 | 0 | 0.0631 | 0.0808 |
| Diabetes | 0.008 | 0.0028 | 2.91 | 0.004 | 0.0027 | 0.0139 |
| High Cholesterol | -0.004 | 0.0024 | -1.76 | 0.078 | -0.0091 | 0.0005 |
| High blood pressure | -0.003 | 0.0025 | -1.35 | 0.177 | -0.0081 | 0.0015 |
| Physical activity | -0.029 | 0.0024 | -12.08 | 0 | -0.0342 | -0.0246 |
| Smoking | 0.014 | 0.0026 | 5.34 | 0 | 0.0088 | 0.0190 |
| Excessive alcohol use | -0.003 | 0.0069 | -0.44 | 0.66 | -0.0165 | 0.0105 |
| Constant | 1.395 | 0.1243 | 11.22 | 0 | 1.1509 | 1.6381 |

Number of obs=11,253, adjusted R-squared=0.1945

**References**

1. Manly, J.J. et al. Estimating the prevalence of dementia and mild cognitive impairment in the US. *JAMA Neurol.* **79,** 1242-1249 (2022).

2. Hurd, M.D., Martorell, P., Delavande, A., Mullen, K.J. & Langa, K.M. Monetary costs of dementia in the United States. *N. Engl. J. Med.* **368,** 1326–1334 (2013).

3. Maddala, G.S. *Limited-Dependent and Qualitative Variables in Econometrics.* (Cambridge University Press, 1983).

1. United Nations Educational, Scientific and Cultural Organization (UNESCO). International Standard Classification of Education (UNESCO, 1997).
2. Folstein, M.F., Folstein, S.E. & McHugh, P.R. Mini-mental state. A practical method for grading the cognitive state of patients for the clinician. *J. Psychiatr. Res.* **12,** 189-198 (1975).
3. Crum, R.M. Population-Based Norms for the Mini-Mental State Examination by Age and Educational Level. *JAMA* **269,** 2386-2391 (1993).
4. Brandt, J., Spencer, M. & Folstein, M. The telephone interview for cognitive status. *Neuropsychiatry Neuropsychol. Behav. Neurol.***1,**111-117 (1988).
5. Morris, J.C. et al. The Consortium to Establish a Registry for Alzheimer’s Disease (CERAD). Part I. Clinical and neuropsychological assessment of Alzheimer’s disease. *Neurology* **39,** 1159-1165 (1989).
6. Ofstedal, M.B., Fisher, G.G. & Herzog, A.R. *Documentation of Cognitive Functioning Measures in the Health and Retirement Study* (Institute for Social Research, University of Michigan, 2005).
7. McArdle, J.J., Fisher, G.G. & Kadlec, K.M. Latent variable analyses of age trends of cognition in the Health and Retirement Study, 1992-2004. *Psychol Aging* **22,** 525-545 (2007).

11. Goodglass, H., Kaplan, E. & Barresi, B*. The Assessment of Aphasia and Related Disorders*. (Lippincott Williams & Wil9kins, 2001).

12. Kertesz, A. *Western Aphasia Battery*. (The Psychological Corporation, 1982).

13. Thurstone, L.L. *Primary Mental Abilities*. (University of Chicago Press, 1938).

14. Lowery, N., Ragland, D., Gur, R.C., Gur, R.E. & Moberg, P.J. Normative Data for the Symbol Cancellation Test in Young Healthy Adults. *Appl Neuropsychol*.**11,** 216-219 (2004).

15. Agrigoroaei, S. & Lachman, M.E. Cognitive Functioning in Midlife and Old Age: Combined Effects of Psychosocial and Behavioral Factors. *J. Gerontol. B Psychol. Sci. Soc. Sci.* **66B,** i130-i140 (2011).

16. Prince, M. et al. A brief dementia screener suitable for use by non-specialists in resource poor settings—the cross-cultural derivation and validation of the brief Community Screening Instrument for Dementia. *Int. J. Geriatr. Psychiatry* **26,** 899-907 (2011).

17. Wechsler, D. *Wechsler Memory Scale-Revised*. (The Psychological Corporation, 1987).

18. Scherr, P.A. et al. Correlates of Cognitive Function in an Elderly Community Population. *Am J Epidemiol*. **128,**1084-1101 (1988).

19. Rosen, W.G., Mohs, R.C. & Davis, K.L. A new rating scale for Alzheimer’s disease. *Am. J. Psychiatry* **141,** 1356-1364 (1984).

20. Smith, A. *Symbol Digit Modalities Test*. (Western Psychological Services, 1982).

21. Fisher, G.G., McArdle, J.J., McCammon, R.J., Sonnega, A. & Weir, D.R. *New Measures of Fluid Intelligence in the HRS* (Institute for Social Research, University of Michigan, 2013).

22. Raven, J. The Raven’s Progressive Matrices: Change and Stability over Culture and Time. *Cogn. Psychol*. **41,**1-48 (2000).

23. Raven, J. The Raven Progressive Matrices: A Review of National Norming Studies and Ethnic and Socioeconomic Variation Within the United States. *J. Educ. Meas.* **26,** 1-16 (1989).

24. Raven, J. *Manual for Raven’s Progressive Matrices and Vocabulary Scales*. Research Supplement No. 1: The 1979 British Standardisation of the Standard Progressive Matrices and Mill Hill Vocabulary Scales, Together with Comparative Data from Earlier Studies in the UK, US, Canada, Germany, and Ireland. (Oxford Psychologists Press, The Psychological Corporation, 1981).

25. Reitan, R.M. *Trail Making Test: Manual for Administration and Scoring*. (Reitan Neuropsychology Laboratory, 1992).

26. Ricker, J.H. & Axelrod, B.N. Analysis of an Oral Paradigm for the Trail Making Test. *Assessment* **1,** 47-51 (1994).

27. Jorm, A.F. A short form of the Informant Questionnaire on Cognitive Decline in the Elderly (IQCODE): development and cross-validation. *Psychol Med.* **24,** 145-153 (1994).

28. Blessed, G., Tomlinson, B.E. & Roth, M. The Association Between Quantitative Measures of Dementia and of Senile Change in the Cerebral Grey Matter of Elderly Subjects. *Br. J. Psychiatry* **114,** 797-811 (1968).

29. Prince, M. et al. The protocols for the 10/66 dementia research group population-based research programme. *BMC Public Health* **7,** 165 (2007).

30. Hall, K.S. et al. The development of a dementia screening interview in two distinct languages. *Int. J. Methods Psychiatr. Res.* **3,** 1-28 (1993).
